# Molecular characterization of senescence marker protein-30 gene promoter: Identification of repressor elements and functional nuclear factor binding sites

**DOI:** 10.1186/1471-2199-9-43

**Published:** 2008-04-29

**Authors:** Bandita Rath, Ravi S Pandey, Priya R Debata, Naoki Maruyama, Prakash C Supakar

**Affiliations:** 1 Institute of Life Sciences, Nalco Square, Chandrasekharpur, Bhubaneswar, 751023, India; 2 Department of Molecular Pathology, Tokyo Metropolitan Institute of Gerontology, 35-2 Sakaecho, Itabashi-ku, Tokyo, 173-0015, Japan

## Abstract

**Background:**

Senescence marker protein-30 (SMP30), whose expression declines during aging in rat liver, has been proposed as an important aging marker. Besides apoptosis, SMP30 also protects cells against various other injuries by enhancement of membrane calcium-pump activity. The mechanism of this differential gene expression mechanism is not known. DNA-protein interactions, mutation analysis and luciferase reporter assay studies have been performed to elucidate the mechanism of transcriptional regulation of SMP30 gene.

**Results:**

We have characterized up to -2750 bp of the promoter by DNA-protein interactions studies. Twenty eight transcription factor binding sites have been identified by DNase I footprinting and electrophoretic mobility shift assay (EMSA). Transient transfection of 5' and 3' -deleted promoter-reporter constructs and luciferase assay illustrated the region between -128/+157 bp is sufficient to drive promoter activity. We have mapped an essential regulatory region between -513 to -352 bp which causes a drastic decline of reporter activity. This region contains CdxA, GATA2 and SRY transcription factor binding sites. Individual mutation of these three sites showed increase in reporter activity. Mutation in SRY site (-403/-368) showed maximum increase in reporter activity among these three sites. Therefore, we suggest that SRY like protein may be acting as a strong repressor of SMP30 gene along with CdxA and GATA-2. We also report that mutation of both Sp1 (172/-148 bp) and a C/EBPβ (-190/-177 bp) transcription binding site located adjacent to each other on SMP30 gene promoter, causes a significant enhancement in reporter activity than individual mutation, thus may be causing the repression of SMP30 promoter activity.

**Conclusion:**

These studies provide novel insights into the mechanism that regulate SMP30 gene expression.

## Background

Senescence marker protein-30 (SMP30), a 34 kDa protein, is preferentially expressed in hepatocytes and renal tubular epithelia. SMP30 is unique in that, its expression is maintained at a high level throughout the tissue maturation process, then decreases in an androgen-independent manner during senescent stages in both sexes [1,2]. Analysis of murine genomic clone revealed that SMP30 is organized into seven exons and six introns spanning approximately 17.5 kb. The full length cDNA fragment (1.6 kb) contains an open reading frame of 897 bp encoding 299 amino acids. Cloned SMP30 promoter is approximately 3 kb in length and up to -1.5 kb of upstream promoter region has been sequenced [2]. We have further sequenced upstream region -3001 to -1502 bp, and the sequence is available from the NCBI database under the accession number EU251064. SMP30 knockout mice though are viable and fertile have reduced body weight and life span. SMP30 deficiency in mice causes an accumulation of neural lipids and phospholipids in liver and shortens the life span [3]. SMP30 plays an important role in maintaining calcium homeostasis as it blunts down cell death caused by intracellular accumulation of calcium by enhancing plasma membranes calcium pumping activity [1]. It also plays a profound role in rescuing cells from cellular injuries such as apoptosis and hypoxia [4]. Besides, SMP30 functions as gluconolactonase in L-ascorbic acid biosynthesis, and its knockout mice are prone to scurvy [5]. Recently, we have also reported the alteration of SMP30 expression in hyperthyroidism [6]. Considering the immense importance of SMP30 in aging and in general physiology of an organism, it is highly essential to understand the mechanism of SMP30 gene expression. Regulation of gene expression at transcriptional level is mediated by the interaction of trans-acting factors with *cis*-acting DNA sequences on the promoter region of the genes. Thus, the cross-talk between trans-acting regulatory factors and *cis*-acting regulatory elements may be important for regulation of SMP30 gene expression. The identification of *cis*-regulatory elements are therefore central and detailed analyses of *cis*-regulatory mechanisms controlling critical transcription factor will be required in order to understand the transcriptional regulation of SMP30 gene. Previously, we reported DNase I footprinting on SMP30 promoter up to -800 bp upstream of the transcription start site and identified eight nuclear factor DNA binding sites in this region excluding -513 to -352 bp [7]. The aim of the present study is to characterize and decipher the mechanism of SMP30 gene expression and regulation. In elucidating the mechanism that endow potent and regulated expression of SMP30, detailed characterization of the promoter is highly desirable. To characterize SMP30 promoter we carried out DNA-protein interaction study by DNase I footprinting studies from -800 bp to -2750 bp and electrophoretic mobility shift assay. In this region about twenty eight putative transcription factor binding sites have been identified of which ten transcription factor binding sites were confirmed by competitive EMSA. Further, to access the transcriptional mechanism of SMP30 gene expression and regulation, we have carried out for the first time 5' and 3' serial deletion of SMP30 promoter and subsequently cloned into luciferase reporter vector. Transient transfection and luciferase assay illustrated the region of SMP30 promoter between -128/+157 bp (Luc-6), having significant promoter activity. Progressive deletion study confirmed the presence of a repressor element between -513 bp to -352 bp. DNase I footprinting assay was carried out to chalk out the repressor elements, which revealed the presence of three DNase I protected sites. Analysis of these sequences with TFSEARCH showed the binding of CdxA, GATA2 and SRY transcription factors. Transient transfection of individual site-directed mutated constructs into RAG cells and luciferase assay showed an increase in reporter activity for all the three mutated constructs.

Since mutation of SRY region (-403/-368) showed maximum reporter activity, we suggest SRY along with CdxA and GATA-2 may be acting as a major negative regulator of this gene. Binding of SRY, GATA-2 and CdxA to their respective sites were confirmed by competitive EMSA. Another interesting feature of SMP30 gene promoter is location of Sp1 and C/EBPβ transcription factor binding sites adjacent to each other. Here, we also report that though the presence of these two transcription factor binding sites to minimal promoter region (Luc-5) did not show any significant change in reporter activity as compared to Luc-6, but mutation of both the transcription factor binding site enhanced the reporter activity significantly by 23%. This suggests either direct or indirect interaction between Sp1 and C/EBPβ occurs at transcriptional level in presence of other regulatory factor in SMP30 promoter which causes repression in SMP30 gene promoter activity.

## Results

### Identification of DNase I protected sites on SMP30 promoter

**Figure 1:**
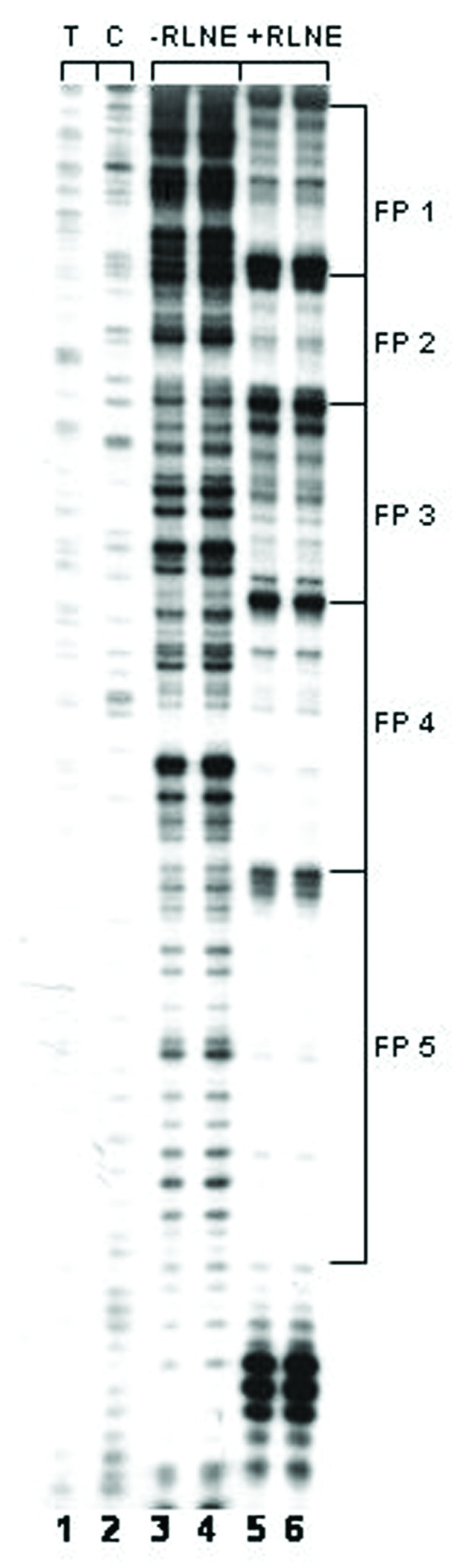
**DNase I footprinting analysis of SMP30 promoter between -1208 bp to -777 bp region: *Lane 1–2*, represent sequencing ladder T and C; *Lane 3–4*, represent DNA treated with DNase I in absence of RLNE; *Lane 5–6*, represent DNA treated with DNase I in presence of 50 μg RLNE.** The DNase I protected sites are marked on right site.

**Figure 2:**
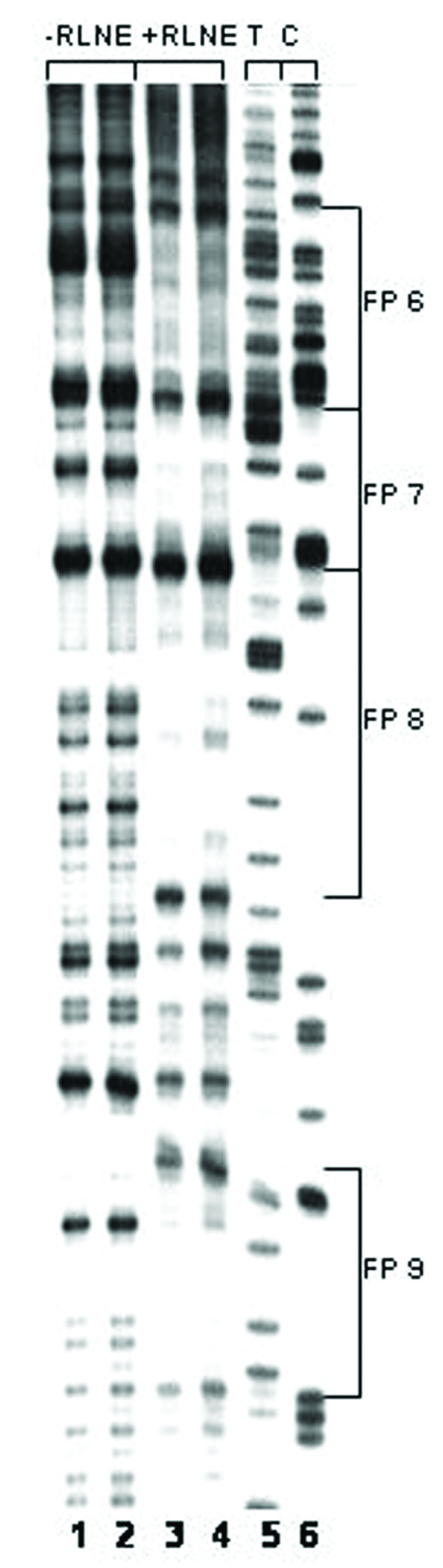
**DNase I footprinting analysis of SMP30 promoter between -1491 bp to -1205 bp respectively: *Lane 1–2 *represent DNA treated with DNase I in absence of RLNE.***Lane 3–4*, represent DNA treated with DNase I in presence of 50 μg RLNE and *Lane 5–6*, represent sequencing ladder T and C.

**Figure 3:**
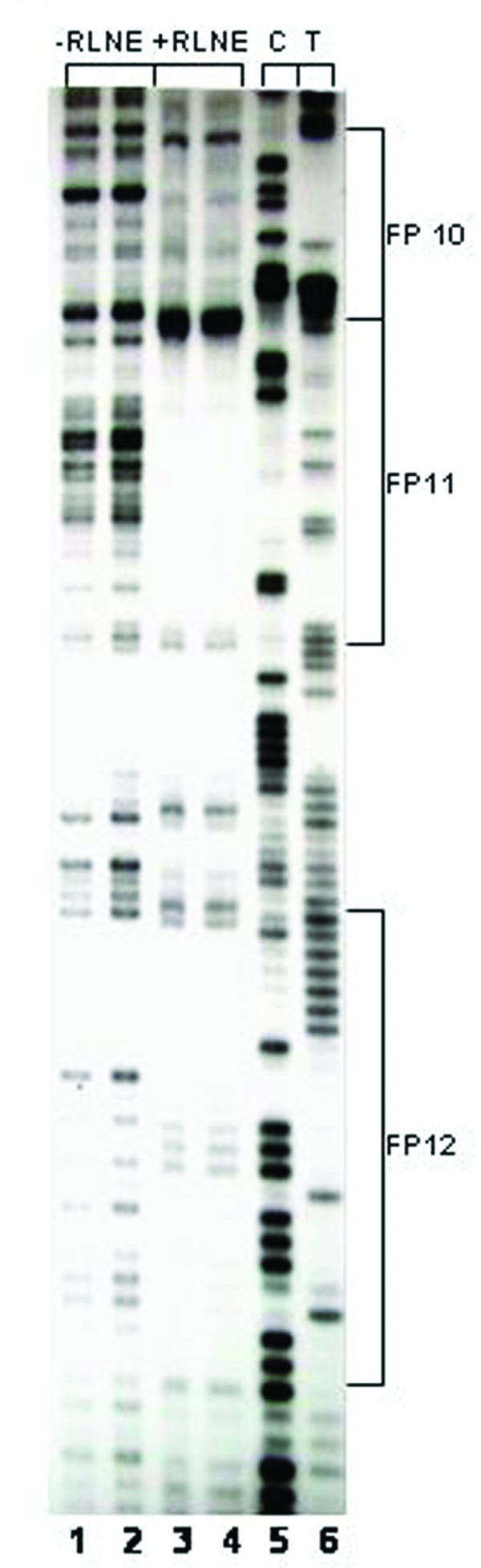
**DNase I footprinting analysis of SMP30 promoter between -2028 to -1626 bp respectively: *Lane 1–2 *represent DNA treated with DNase I in absence of RLNE.***Lane 3–4*, represent DNA treated with DNase I in presence of 50 μg RLNE *Lane 5–6*, represent sequencing ladder C and T.

We have previously identified eight transcription factor binding sites within 0.8 kb mouse SMP30 promoter fragment by DNase I footprinting and EMSA [7]. In this study we further investigated the transcription factor binding sites by DNase I footprinting assay on SMP30 promoter region between -2750/-777 bp using rat liver nuclear extract. The Primers used for footprinting study of the above mentioned regions are shown in table 1. Within this region, twenty eight DNase I protected sites were identified (Table 2 and 3) and a representative of three DNase I footprinting sites of the regions -1208/-777 bp, -1491/-1205 bp and -2028/-1626 bp are shown (Figure 1, 2 and 3).

**Table 1:** Primers used for footprinting from -777 to -2750 kb.

**Serial No**	**Region Amplified**	**Primers: Sense (SS) and antisense (AS)**
1	-2008 to -777	SS: CAGCATTCCTGGTAGAAACAGGTCC
2	-2008 to -777	AS: GTCCTACACATGGGTGGGCAAATG
3	-1276 to -1039	SS: GCTTCCCAGAGTTCGGCCATTGTTG
4	-1276 to -1039	AS: GTCTTGCAAGCGATGTGTGTGG
5	-1491 to -1205	SS: CCCTTCCCAAGGTTCTCTGC
6	-1491 to -1205	AS:GGTTTTCCCATTGTGACGACGTCGG
7	-1695 to -1391	SS: CACTTGCTTTAACTCCTGCAGC
8	-1695 to -1391	AS: GCTTCTTCATCTTACCCACC
9	-2028 to -1626	SS: GACACACCAGGTGAGCACTGTAC
10	-2028 to -1626	AS: GGTAACTGGAAGTACCCAGC
11	-2190 to -1865	SS: CAAGGCCAGCATGGACTGC
12	-2190 to -1865	AS: GAAGACCTTGGTGGCAGCAG
13	-2448 to -2112	SS: GGTATGCATGCATGCAGTGC
14	-2448 to -2112	AS: GAGCCAATCACCTCCAGGTG
15	-2750 to -2283	SS: GAACGGCAAAGTTAGTATGAGGCC
16	-2750 to -2283	AS: GAGACAGTCCTCAAGTAGCCTGC

**Table 2:** DNase 1 protected sites.

**Footprint**	**DNase 1 protected region**	**Transcription factor**
**FP 4**	TGTGGGTTAAG*CTATTGCAAAACTC*CAACATCTGATCTTGGGGCTT	C/EBP β
**FP 5**	ACCCCTCCAC*ACACATCGCT*TGCAAGACAAACTGTGGGTT	GATA1
**FP 6**	C*ACCCCAA*TCCGGCTGAGACTGCTCTGTGAGTAGC	AML-1A
**FP 8**	CTTGGTGGGTAAGATGAAGAA*GCTAGATTTGG*GCGAAGGC	GATA1
**FP 10**	TTTGCAAGCGTTGGCCTGCTGCCACCAAGGTCTTCCC	Novel
**FP 11**	TAAACCA*AATCAAA*TAAAGGCATTTTTCTTCCCCTTCC	SRY

**Table 3:** DNase 1 protected sites between -777 to -2750 (data not shown)

**Footprint**	**DNase 1 protected region**	**Transcription factor**
**-953 to -913**	GGGCCA*ATTTTTA*ACAGCCAATGAAAATGGCAAATGCTACACA	CdxA
**-995 to -954**	*GGGGCTTATCT*ACGATTGATAGCATGAAGC	GATA-X
**-1123 to -1088**	CCACCAGTTTGCAGCCAGAATTCCTGG*TAGAAAC*AG	CdxA
**-1374 to -1351**	CCCCTGGGAAGCTAGA*TTTGTTC*A	SRY
**-1440 to -1419**	CCCTG*TGAATAACCG*GGACAGG	HSF2
**-1546 to -1520**	CCCCGGGCCGGCGGCT*ACCTATCTGC*C	GATA2
**-1584 to -1550**	AGAAGGGTGAGCCCT*CAGGATCGCT*AGTCTCTGCC	GATA2
**-1621 to -1587**	GCTG*AAAAATG*AAAGGACAGCGTGGGCACCCGTAG	Cdxa
**-1983 to -1956**	G*GCTATGTCATTTAG*AATCGTTTATTCC	Oct-1
**-1994 to -1981**	TAGC*TATGTTT*GGC	SRY
**-2076 to -2051**	*CCTATAAAAT*AAAGGGAAAAGAACACC	TATA
**-2111 to -2082**	ACCTGGA*GGTGATTGGC*TCTGAGTTTCACC	GATA1
**-2134 to -2109**	GGGGA*GTGAGTCAG*TGGTAGTGCACC	Ap1
**-2167 to -2141**	TGAAACTGCCAAGA*AATAATG*TCTTAG	CdxA
**-2384 to -2347**	TAAATAGGTTTTTAAAAAGAA*AAAGAAA*AAATGGGGCA	SRY
**-2414 to -2381**	AAGCAAAGCCCTCACACA*CATTAAA*ATGAGTAAA	CdxA
**-2491 to -2449**	GAGATGACAATTACATCA*AATAATA*AAAGTTATATTTACATCA	CdxA
**-2604 to -2562**	TGGTGA*GCTTATTTAATC*TCAGAGACTGAAAACATTCTAGGCC	HNF-3b
**-2675 to -2646**	GCTTGGCGA*GGAGTTT*TAACCCAGAACAGC	SRY
**-2691 to -2654**	AGAAGCCAGA*TTGAATCAG*TTGCTTGGCGAGGAGTTTTAACCC	AP1
**-2704 to -2691**	CCAAGAGAAGAAGCC	Novel
**-2721 to -2703**	GAG*CAATTCAG*GAGAGGCC	Nkx-2.5

**Table 4:** Primers used for 5' and 3' deletion.

Luc-1 (SS)	ACA*GGTACC*CAAATGCTACAGCGCTGG
Luc-2 (SS)	ACA*GGTACC*AATGTCTACTGGGGTAG
Luc-3 (SS)	ACA*GGTACC*CATGCAAGGAAGCAAG
Luc-4 (SS)	ACA*GGTACC*CCTCATACCTGCCATTATC
Luc-5 (SS)	ACA*GGTACC*TACCAAGCCTCTGGCTG
Luc-6 (SS)	ACA*GGTACC*GAATGAGGGAGAGGTG
Luc-SMP-XhoI (AS)	ACA*CTCGAG*GCAAGACAGGAGGTGATTG
Luc-Exon-SMP-Xho1	ACA*CTCGAG*CGTCTTCAGTCAACTTACC

### Confirmation of identified transcription factor binding site through electrophoretic mobility shift assay (EMSA)

**Figure 4:**
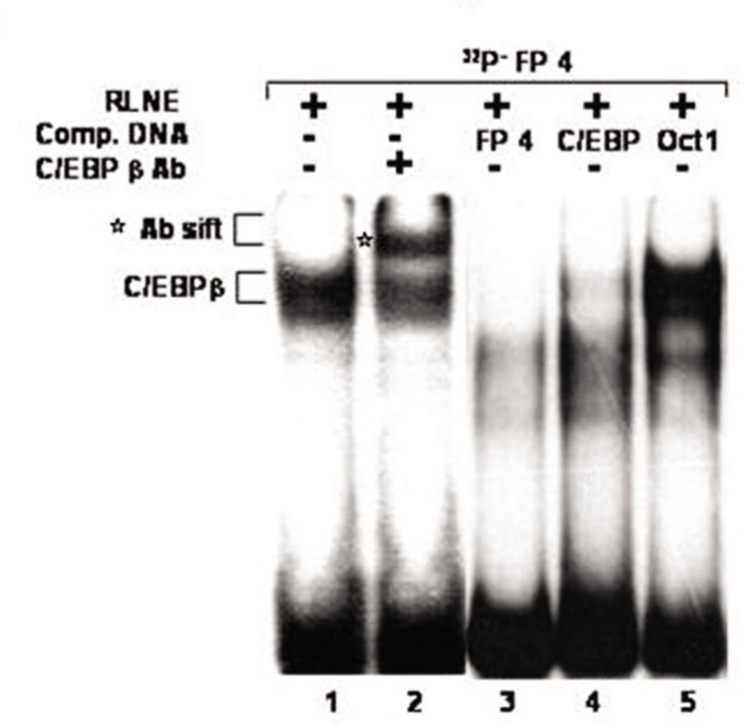
**Electrophoretic mobility shift assay to confirm the binding of C/EBP transcription factor to DNase I protected site FP 4: *Lane 1*, labeled FP 4 oligonucleotide duplex with 6 μg RLNE; *Lane 2 *C/EBPβ antibody; *Lane 3–5*, describe the competition with 100 fold molar excess of unlabeled homologous self, C/EBP consensus, and nonspecific Oct 1 oligonucleotide duplex respectively.** Antibody shift is seen with C/EBPβ antibody.

**Figure 5:**
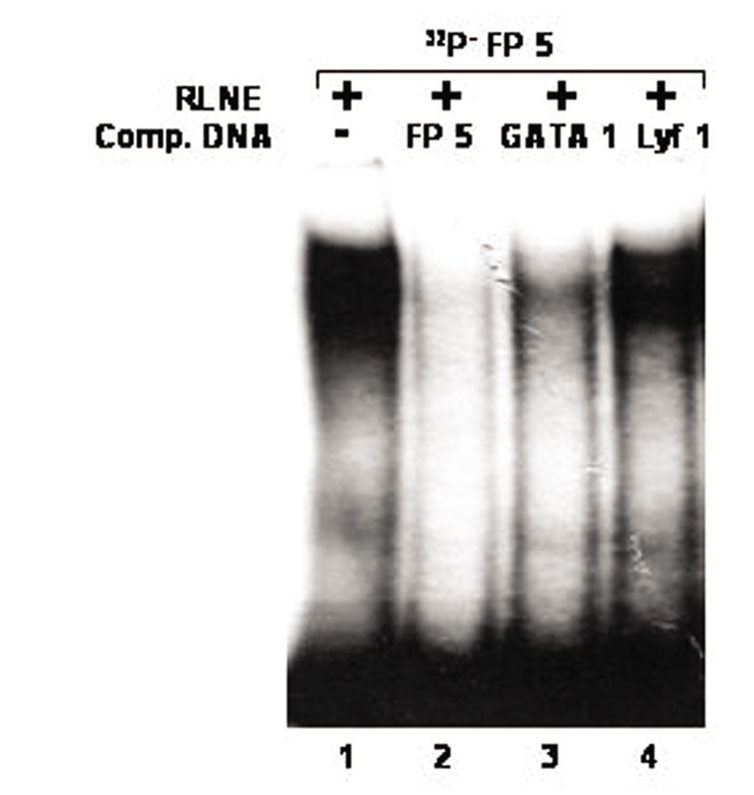
Electrophoretic mobility shift assay to confirm the binding of GATA1 transcription factor to DNase I protected site FP 5: *Lane 1*, labeled FP 5 oligonucleotide duplex with 6 μg RLNE; *Lane 2–4*, describe the competition with 100 fold molar excess of unlabeled homologous self, GATA1 consensus and Lyf-1 consensus.

**Figure 6:**
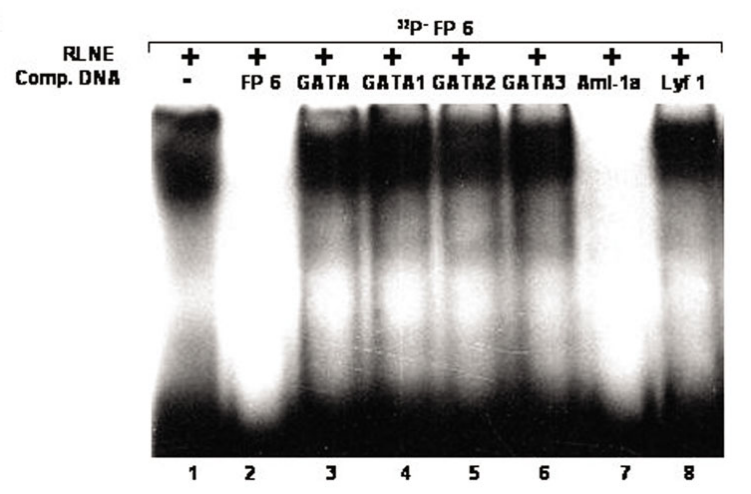
Electrophoretic mobility shift assay to confirm the binding of Aml-1a transcription factor to DNase I protected site FP 6: *Lane 1*, labeled FP 6 oligonucleotide duplex with 6 μg RLNE; *Lane 2–8*, describe the competition with 100 fold molar excess of unlabeled homologous self, GATA consensus, GATA1 consensus, GATA2 consensus, GATA3 consensus, Aml-1a and nonspecific Lyf-1 oligonucleotide duplex respectively.

**Figure 7:**
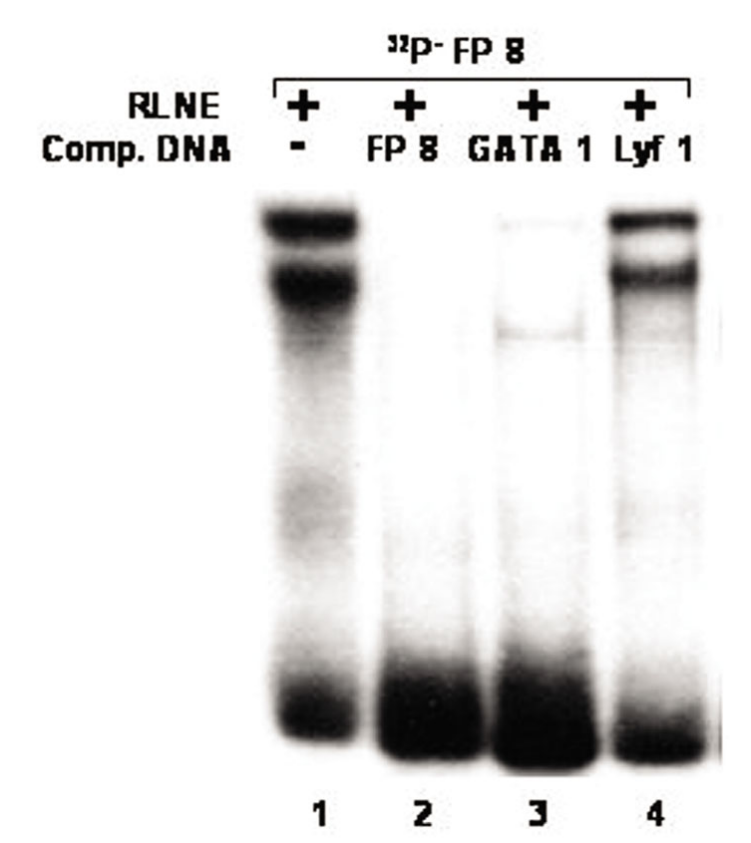
Electrophoretic mobility shift assay to confirm the binding of GATA1 transcription factor to DNase I protected site FP 8: *Lane 1*, labeled FP 8 oligonucleotide duplex with 6 μg RLNE; *Lane 2–4*, describe the competition with 100 fold molar excess of unlabeled homologous self, GATA1 consensus, and Lyf-1 consensus.

**Figure 8:**
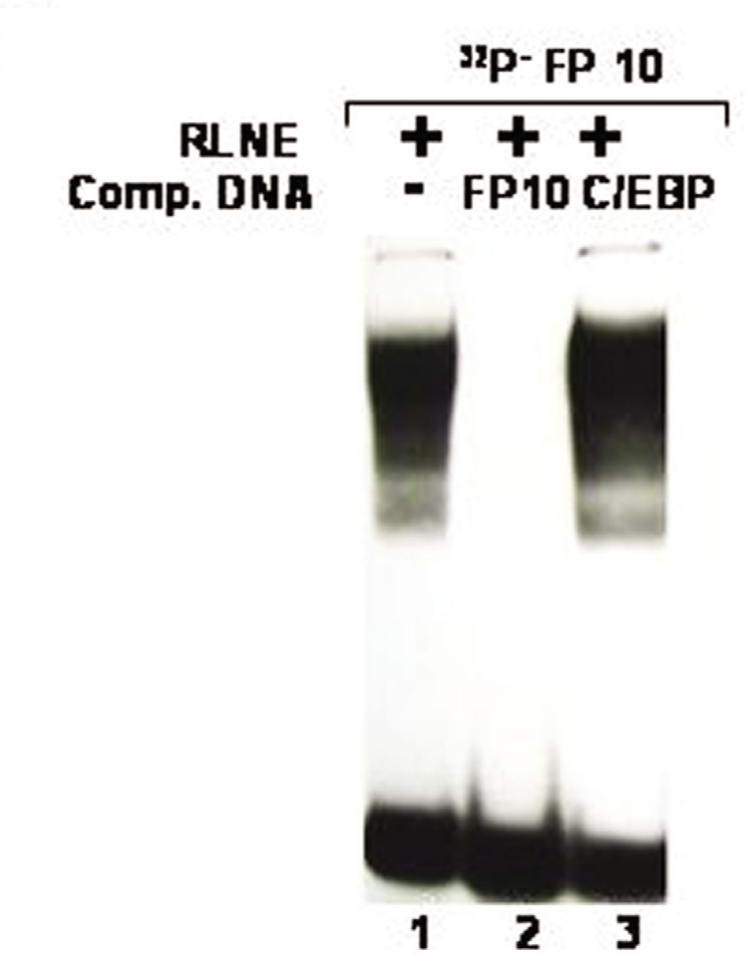
**Electrophoretic mobility shift assay of DNase I protected site FP 10: *Lane 1*, labeled FP 10 oligonucleotide duplex with 6 μg RLNE; *Lane 2–3*, describe the competition with 100 fold molar excess of unlabeled homologous self, and non specific C/EBP consensus.** Binding of no transcription factor was observed in TFSEARCH data base to this site.

**Figure 9:**
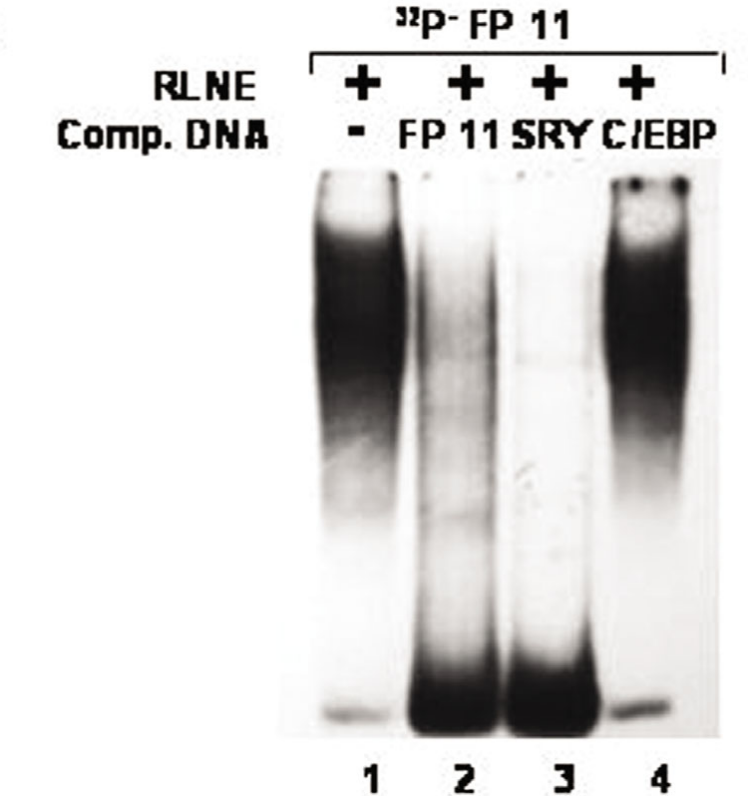
Electrophoretic mobility shift assay to confirm the binding of SRY to DNase I protected site FP 11: *Lane 1*, labeled FP 11 oligonucleotide duplex with 6 μg RLNE; *Lane 2–4*, describe the competition with 100 fold molar excess of unlabeled homologous self, SRY and non specific C/EBP consensus.

All the DNase I protected sequences were analyzed in transcription factor data base (TFSEARCH, Japan) which revealed binding sites sequence homology to multiple transcription factors. To demonstrate the specificity of transcription factors binding sites to the DNase I protected regions, we carried out EMSA and/or supershift assay. We synthesized oligonucleotides (both strands) corresponding to the protected sites (Table 2) and prepared radiolabeled duplexes for EMSA studies. Five DNase I protected sites were identified in the region -1208/-777 bp of which FP 4 and FP 5 have been confirmed by EMSA (Figure 4 and 5). To FP 4 site binding of C/EBPβ was confirmed by competition with cold C/EBP oligonucleotide and also by C/EBPβ antibody shift experiments. Binding of GATA-1 to FP 5 is confirmed by competition experiments. Four DNase I protected sites were detected in the region -1491/-1205 bp. Though TFSEARCH revealed the binding GATA-3, GATA-1, GATA-2 and AML-1a in order of decreasing binding affinity, only AML-1a competed with FP 6 site (Figure 6). Similarly TFSEARCH revealed the binding of Lyf-1 and GATA-1 to FP8, only cold GATA-1 oligonucleotide competed with FP 8 but not Lyf-1 thus confirming the binding of GATA-1 to FP8 (Figure 7). Three DNase I protected sites were detected in -2028/-1626 bp region. Binding of no transcription factor up to 80% was observed in TFSEARCH to FP 10, thus it may be a novel transcription factor binding site (Figure 8). Binding of SRY to FP 11 is confirmed by competition studies (Figure 9). Table 3 shows all the DNase I protected sites confirmed by EMSA (data not shown).

### Elucidation of mechanism of SMP30 gene expression and regulation

**Figure 10:**
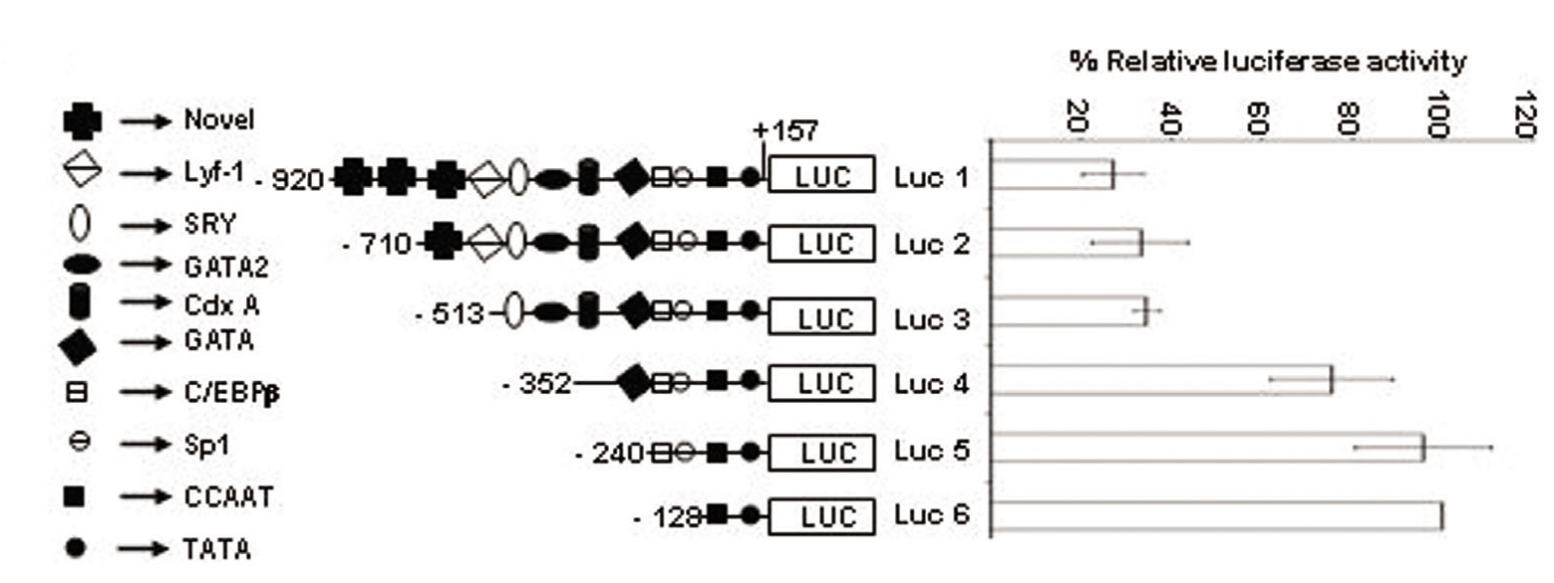
**Relative luciferase activity of different 5' -serially deleted SMP30 promoter-reporter constructs were transfected in RAG cells.** The results were obtained after normalization with ß-galactosidase activity. All transfections were repeated in duplicates and the results are expressed as the mean of five different experiments ± S.D. On left, a schematic representation of all the 5' -deleted luciferase constructs used for transfection is depicted. Approximate locations of transcription factor binding sites are shown.

**Figure 11:**
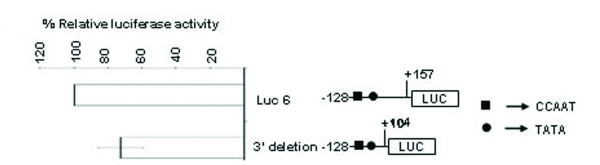
**Luciferase analysis of 3' -deleted Luc 6 constructs as compared to wild type Luc 6 construct showing ~ 28% reduction of reporter activity. **All transfections were repeated in duplicates and the results are expressed as the mean of two different experiments ± S.D. On right, a schematic representation of 3' deleted luciferase construct used for transfection is depicted along with wild Luc 6. Approximate locations of transcription factor binding sites are shown.

To elucidate the mechanism of SMP30 gene expression, both 5' and 3' deletion constructs were sub-cloned into pGL3 luciferase plasmid and transiently transfected into RAG cells. The 5' -serially deleted constructs are -920/+157(Luc-1), -710/+157(Luc-2), -513/+157(Luc-3), -352/+157(Luc-4), -240/+157(Luc-5) and -128/+157(Luc-6). The 3' -serially deleted constructs are +157/-128 and +104/-128. The expression pattern of 5' -serially deleted constructs is shown in figure 10. The 5' -deleted -128/+157 bp construct (Luc-6) showed highest reporter activity among others. In order to delineate the basal promoter activity further, we carried out transfection of 3' -deletion construct into RAG cells. The construct -128/+104 bp showed ~ 28% reduction in reporter activity (Figure 11). Thus, the region between -128/+157 bp is essential in determining the promoter activity of SMP30 gene and sequence between +104 bp to +157 bp plays a detrimental role during transcription.

### Identification of SRY, GATA-2 and CdxA like transcription factor as a repressor element present between -513 and -352 bp

**Figure 12:**
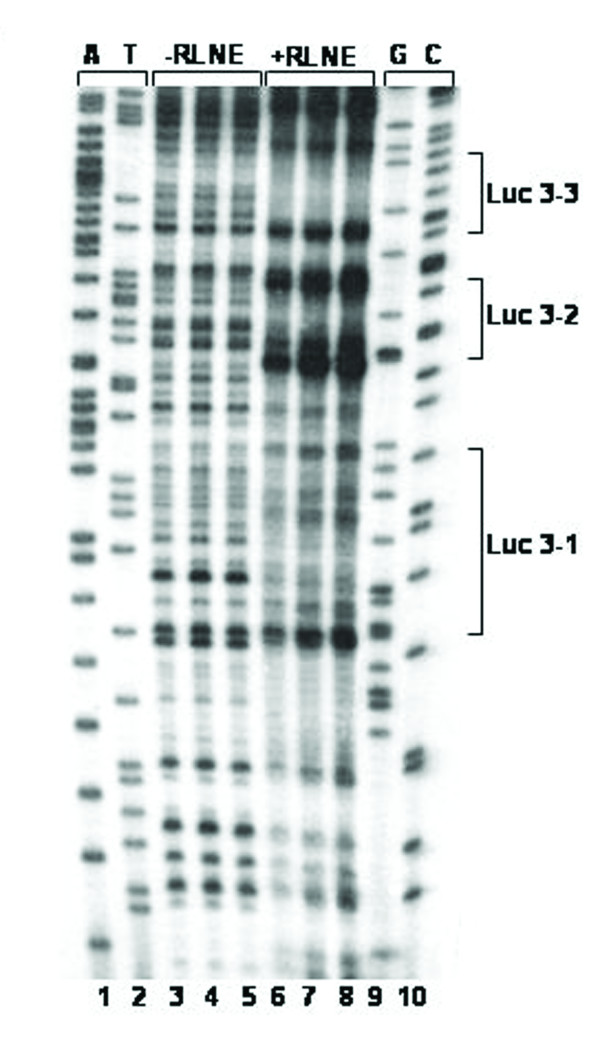
**DNase I footprinting pattern of the repressor region (-513 to -352). ***Lane1,2 and 9,10 *refer to sequencing reaction ladder obtained by taking the same labeled primer used in PCR for DNase I footprinting. *Lane 3,4,5 *represent DNA treated with DNase I in absence of nuclear protein. *Lane 6,7,8 *represent DNA treated with DNase I in presence of 50 μg rat liver nuclear extract (RLNE). The DNase I protected sites are marked on right site and the sequences are given in table 3. DNase I footprinting was carried out with fragments generated by labeled forward primer.

**Figure 13:**
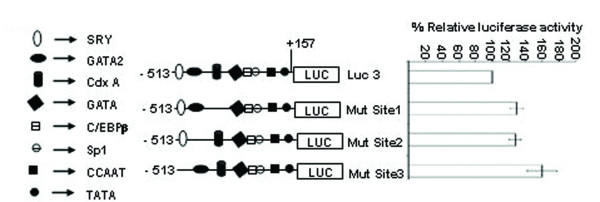
**DNase I protected sites, Luc 3–1, Luc 3–2 and Luc 3–3 were mutated at their core binding sites.** Reporter activity of the three mutated construct along with wild type Luc 3 construct is represented on the right site and the schematic representation of the mutated sites along with wild type Luc 3 on left site. All transfections were repeated in duplicates and the results are expressed as the mean of three different experiments ± S.D Approximate location of the transcription factor binding to wild type Luc 3 and site directed mutated construct are shown. Mutation of site 3 shows a significant (~59%) increase in luciferase activity as compared to wild type Luc3.

**Figure 14:**
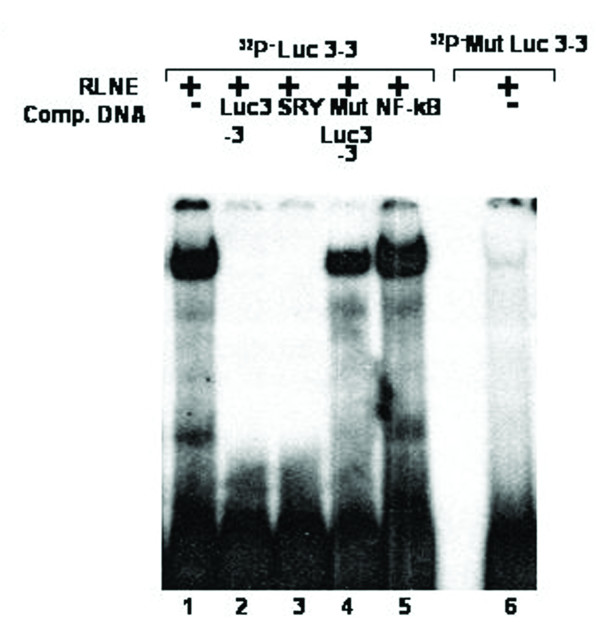
**Electrophoretic mobility shift assay (EMSA) for site Luc 3–3 to confirm the binding of SRY transcription factor.***Lane 1*, labeled oligonucleotide duplex with 6 μg RLNE; *Lane 2–5*, 100 fold molar excess of unlabeled homologous self, SRY consensus, mutated Luc 3–3, nonspecific oligonucleotide duplex (NFkB). *Lane 6*, labeled Mut 3–3 oligonucleotide duplex with 6 μg RLNE.

**Figure 15:**
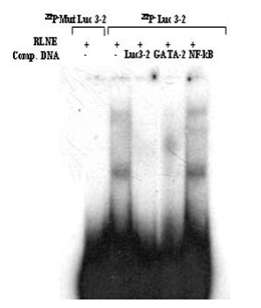
**EMSA for site Luc 3–2 to confirm the binding of GATA-2 transcription factor.***Lane 1*, labeled mutated oligonucleotide duplex with 6 μg RLNE; *Lane 2*, labeled oligonucleotide duplex with 6 μg RLNE; *lane3 -5*, 100 fold molar excess of unlabeled homologous self, GATA-2 consensus, and nonspecific (NFkB) oligonucleotide duplex (NFkB).

The reporter assay of 5' -serially deleted constructs showed a drastic decline (~41%) of activity of Luc-4 (-352/+157 bp) as compared to Luc-3 (-513/+157 bp). DNase I footprinting study of the region between -513 bp and -352 bp revealed three distinct DNase I protected sites such as Luc 3–1, Luc 3–2 and Luc 3–3 (Figure 12). TFSEARCH revealed the binding of CdxA to Luc 3–1, GATA-2 to Luc 3–2 and SRY to Luc 3–3. To pin point the transcription factor which act as a repressor we prepared site directed mutated constructs for all the three sites. Transient transfection of these mutated constructs was carried out along with wild type Luc 3 (-513/+157) (Figure 13). Mutation of Luc3-1 leads to increase in reporter activity by 29%, Luc3-2 by 27% and Luc 3–3 site leads to maximum increase in reporter activity about 59%. This result revealed SRY, CdxA and GATA-2 as the major repressor elements. To further establish this fact, we did EMSA study using both wild type and mutated oligonucleotide of Luc3-3 site. The wild type Luc3-3 oligonucleotide competed with SRY specific cold oligonucleotide consensus, but mutated 3–3 site did not show any binding, thus establishing the binding of SRY to this site (Figure 14). The competitive EMSA using radiolabeled Luc3-2 oligonucleotide showed 100% competition with GATA-2 (Figure 15), while EMSA using radiolabeled Luc 3–1 which has 85% homology revealed that although there is competition by unlabeled Luc 3–1 oligonucleotide, no significant competition is observed using consensus CdxA site. Considering the high homology to Cdx A consensus binding site, Luc 3–1 site may be interacting with CdxA like transcription factor with slight deviation in binding sequence (data not shown).

### Sp1 and C/EBPβ causes repression of SMP30 promoter activity

**Figure 16:**
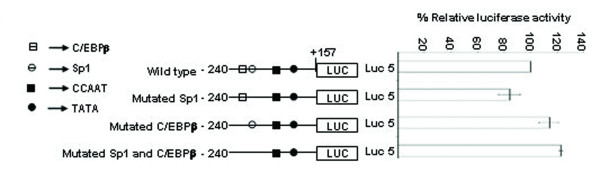
**Reporter activity of site direct mutated Sp1, site direct mutated C/EBP and site direct mutated Sp1 and C/EBP sites reporter constructs along with wild type Luc 5 on right site and the schematic representation of the mutated Sp1 and C/EBP sites along with wild type Luc 5 sites on left site.** All transfections were repeated in duplicates and the results are expressed as the mean of three different experiments ± S.D Approximate location of the transcription factor binding to wild type Luc 5 and site directed mutated construct are shown. A significant decrease (~16%) in reporter activity of mutated Sp1 construct is seen as compared with wild type Luc 5. There is no significant change in reporter activity with mutated C/EBP construct. Double mutation of Sp1 and C/EBP enhance the reporter activity by 23% as compared to wild type Luc 5.

**Figure 17:**
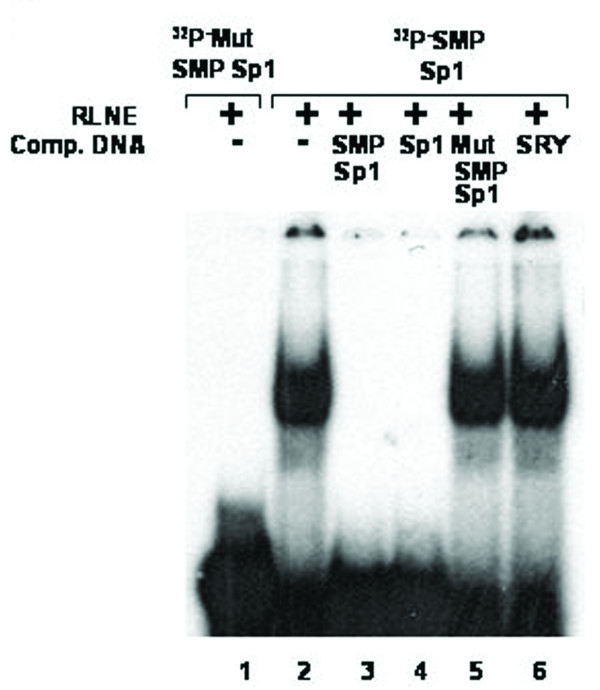
**Electrophoretic mobility shift assay to confirm the binding of Sp1 transcription factor.***Lane 1*, labeled mutated SMP-Sp1 oligonucleotide duplex with 6 μg RLNE; *Lane 2–6*, labeled SMP-Sp1 oligonucleotide duplex with 6 μg RLNE; *Lane 3–6*, describe the competition with 100 fold molar excess of unlabeled homologous self, Sp1 consensus, Mut SMP-Sp1 and nonspecific SRY oligonucleotide duplex respectively.

**Figure 18:**
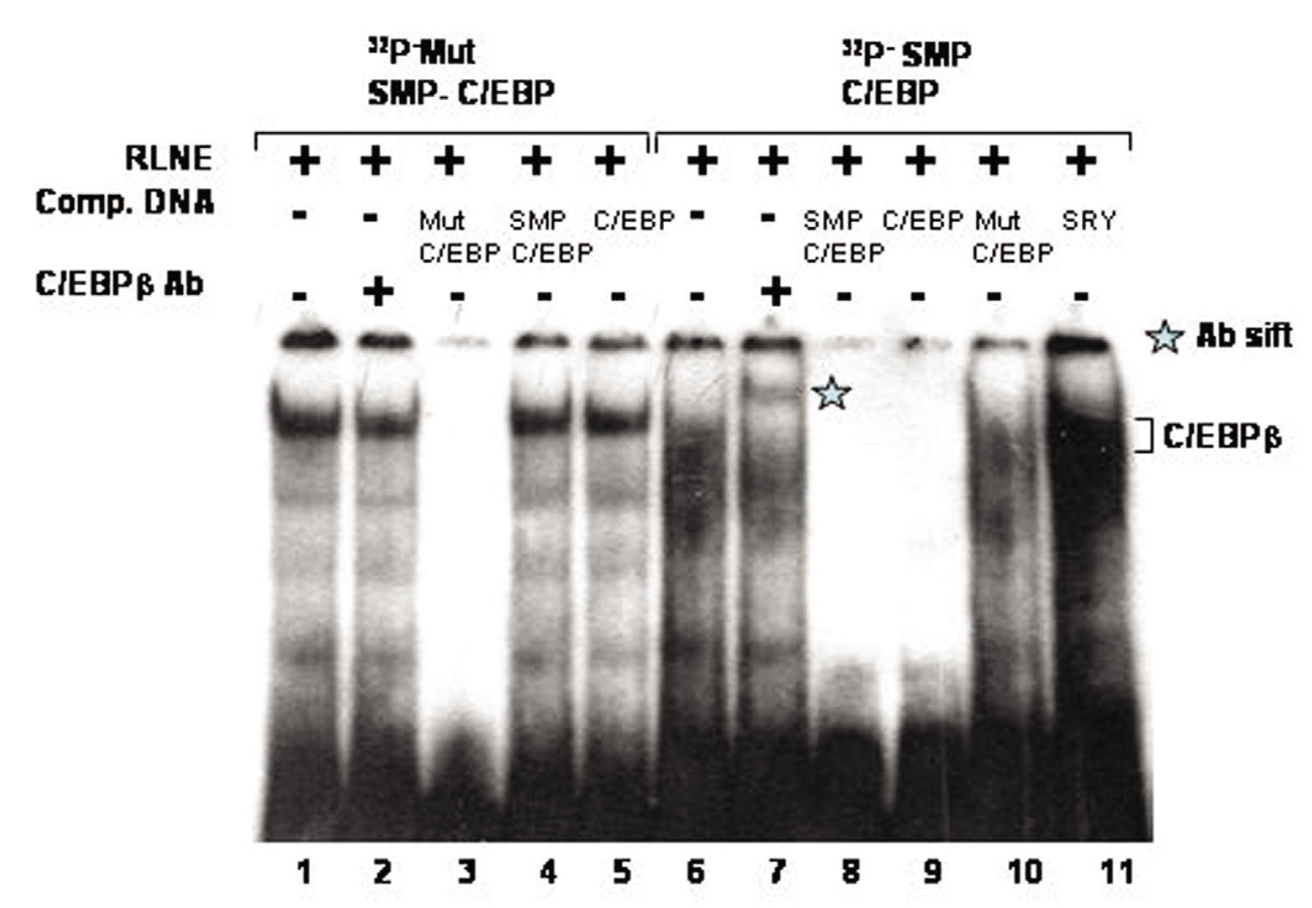
**Electrophoretic mobility shift assay to confirm the binding of C/EBP transcription factor.***Lane 1–5*, labeled mutated C/EBP oligonucleotide duplex with 6 μg RLNE; *Lane 2*, C/EBPβ antibody; *Lane 3–5*, describe the competition with 100 fold molar excess of unlabeled homologous self, SMP-C/EBP and C/EBP consensus. *Lane 6–11*, labeled SMP-C/EBP oligonucleotide duplex with 6 μg RLNE; *Lane 7*, C/EBPβ antibody; *Lane 8–11*, describe the competition with 100 fold molar excess of unlabeled homologous self, C/EBP consensus, Mut SMP-C/EBP and nonspecific SRY oligonucleotide duplex respectively. Antibody shift is seen with SMP-C/EBP only and not with mutated SMP-C/EBP.

SMP30 gene promoter has a Sp1 and a C/EBPβ transcription factor binding site adjacent to each other. Sp1 site spans between -172 to -148 bp and C/EBPβ spans between -190 to -177 bp. Here we report that, presence of these two sites in the minimal promoter region did not cause any significant change in reporter activity (that is, there is no significant change in Luc-5 as compared to Luc-6). But site-directed mutation of both the transcription factor binding site caused a significant increase in reporter activity (~23%) (Figure 5A). Individual mutation of only Sp1 and C/EBPβ did not contribute to any significant change in reporter activity. Mutation of Sp1 site reduced the reporter activity by only 16% and mutation of C/EBPβ lead to enhancement of reporter activity by only 14% (Figure 16). Binding of Sp1 to the region between -172 to -148 bp is confirmed by competitive EMSA done in presence of 100 fold molar excess of cold Sp1 consensus (Figure 17). EMSA was also carried out using labeled mutated Sp1 oligonucleotide, which showed no DNA-protein interaction, thus confirming the inability of Sp1 to bind to the mutated site. Binding of C/EBPβ to the region -190/-177 bp is confirmed by EMSA and antibody shift experiments using C/EBPβ antibody (Figure 5C). EMSA study carried out with labeled mutated C/EBP oligonucleotide also yielded a DNA-protein complex. But this complex is not due to binding of C/EBPβ as confirmed by competition with C/EBPβ consensus and antibody shift experiments (Figure 18).

## Discussion

The multiple biological functions of SMP30 in diverse target cells require its expression to be regulated precisely. It is suggested that the transcriptional regulation of a particular gene is a complex process which usually involves interaction between multiple *cis*-acting regulatory elements and their cognate protein factors [8,9]. A growing list of transcription factors has been shown to function as either transcriptional activator or repressor in different gene promoter. In this study we analyzed the transcriptional regulation of SMP30 gene by DNase I footprinting, EMSA and functional characterization by transient transfection, reporter assay of 5' and 3' -serially deleted promoter reporter constructs and site-directed mutagenesis. We have earlier reported eight nuclear factor binding sites on SMP30 gene promoter [7]. In this report twenty eight new DNase I footprinting sites were identified using rat liver nuclear extract. We also demonstrate that the 5' -flanking regions of SMP30 gene possess a functional promoter when transfected into RAG cells. The results of 5' and 3' -deletion analysis illustrated the region -128/+157 bp possesses significant reporter activity. The presence of a TATA sequence (-29 ATAAAA -25) and a CAAT box (-72 CCAAT -68) were previously reported respective to the transcription start site [2]. Our results suggest that the TATA and CAAT box located between -128 bp and +157 bp plays an important role in determining the promoter activity and sufficient to drive SMP30 gene expression. 3' – deletion from +157 bp to +104 bp resulted in ~28% decrease in basal promoter activity, thus indicating that this region is essential for SMP30 gene expression. An interesting feature of SMP30 promoter is the presence of C/EBPβ binding site adjacent to Sp1 binding site. Sp1 is a ubiquitous DNA-binding protein with three zinc finger at its C-terminal that activates the transcription of many cellular and viral genes [10]. SMP30 promoter possess a Sp1 binding site between -172 bp to -148 bp. C/EBPβ belongs to CCAAT-enhancer-binding protein family of transcription factors, involved in different cellular response like in control of cellular proliferation, growth and differentiation, metabolism, immune response and many others. C/EBPβ binding site spans between -190 bp to -177 bp on SMP30 promoter. This spatial arrangement of C/EBPβ and Sp1 is critical as Sp1 is known to recruit C/EBPβ to cryptic C/EBP site [11]. Presence of these two sites in the minimal promoter region represented as Luc-5 did not show any significant change in luciferase activity as compared to the Luc-6, but mutation of both Sp1 and C/EBPβ significantly enhanced the reporter gene activity to about 23%. Thus, it is reasonable to believe that direct or indirect interaction between Sp1 and C/EBPβ in presence of some other regulatory factor occurs at transcriptional level in SMP30 promoter which causes a repression in SMP30 promoter activity. Transient transfection of 5' -deletion fragments revealed the presence of a repressor element between -513 to -352 bp, as deletion of this region caused 41% decrease in reporter activity. Our DNase I footprinting study showed three putative transcription factor binding sites within this region (Figure 12). In order to confirm the potential repressor among these DNase I protected sites, we carried out site directed mutagenesis studies of these three sites and subsequent transfection along with wild type (Luc-3). This result suggested a significant enhancement of reporter activity of Luc 3–3 mutated fragment by ~59%, Luc3-2 by 27% and Luc 3–1 by 29%. (Figure 13). TFSEARCH indicated the binding of SRY to wild type site Luc 3–3, GATA-2 to Luc 3–2 and CdxA to Luc 3–1 sequences, which is confirmed by competitive EMSA. Earlier reports depicted the tissue specific expression of SRY in testes [12] where it involve in testes determination and differentiation in mammals. Though expression of SRY in substantia nigra of adult male rodents in tyrosin hydroxylase expressing neurons has also been reported but its expression in liver and kidney is still obscure [13]. So the identified transcription factors might be SRY like proteins which bind to a similar binding site as SRY. The affinity of SRY for double-stranded DNA varies with DNA sequence and shares a conserved DNA binding domain (HMG-box) NACAAT [14]. SRY is reported to bind and negatively regulates the androgen receptor gene promoter [15]. We also suggest that GATA-2 and CdxA might be interacting directly or indirectly with SRY to bring about repression of SMP30 gene.

## Conclusion

Transcription factors Sp1, C/EBPβ, SRY, GATA-2 and CdxA, binding within -513 of SMP30 promoter, have significant role in regulation of SMP30 gene expression.

## Methods

### Preparation of nuclear extract

Nuclear extract from liver of adult (5 months) male rats (Fisher 344) were prepared as described previously [16]. Briefly, liver slices were homogenized in 4 volumes (w/v) of ice-cold buffer containing 0.25 M sucrose, 15 mM Tris-HCl (pH 7.9), 16 mM KCl, 15 mM NaCl, 5 mM EDTA, 1 mM EGTA, 1 mM DTT, 0.15 mM spermine, and 0.15 mM spermidine; supplemented with the following protease inhibitors: 0.1 mM PMSF, 2 μg/ml leupeptin, 5 μg/ml aprotonin. After centrifugation for 10 minutes at 2000 × *g*, the pellets were resuspended in 4 volumes of ice-cold buffer (10 mM HEPES; pH 7.9, 1.5 mM MgCl_2, _10 mM KCl, and protease inhibitors). The nuclei were pelleted down by centrifugation for 10 minutes at 4000 × *g*, and resuspended in ice cold buffer of 10 mM HEPES (pH 7.9), 0.75 mM MgCl_2, _0.5 M KCl, 0.5 mM EDTA, 12.5% glycerol and protease inhibitors. After incubation on ice for 30 minutes with continuous agitation, the supernatants containing the nuclear extracts were collected by centrifugation for 30 minutes at 14,000 × *g*, frozen in liquid nitrogen and stored in -70°C until used. All manipulations were carried out at 4°C. Protein concentrations were determined by the Bradford protein assay reagent (Sigma, USA).

### DNase I footprinting

DNase I footprinting was carried out as described before [7]. Briefly, end-labeled DNA fragments (50 fmoles) were incubated with 50 μg of rat liver nuclear extract and 2 μg of poly (dI-dC) in binding buffer containing 10 mM Tris-HCl (pH 7.5), 50 mM NaCl, 1 mM EDTA, 1 mM DTT, 5% glycerol at room temperature for 30 minutes. Subsequent to binding reaction 7.5 mM MgCl_2 _and 5 mM CaCl_2 _were added and samples were incubated at room temperature with DNase I (0.25 U, Roche, USA) for 60 s. Ten to twenty folds less DNase I was used for control experiments without nuclear extracts. DNase I enzyme digestion was stopped by the addition of an equal volume of 1% SDS, 20 mM EDTA, 400 mM NaCl, 100 μg/ml yeast tRNA and 200 μg/ml proteinase K. Following incubation at 45°C for 60 minutes, samples were extracted twice with phenol/chloroform, precipitated with ethanol and electrophoresed on 6% polyacrylamide sequencing gel. After electrophoresis, gels were dried on Whatman filter paper and autoradiographed. Primers used for generating end labeled DNA fragments for footprinting of the region -513/-352 bp are: 5' -GCCTCATGCAAGGAAGCAAG-3' SS and 5' -GATAATGGCAGGTATGAGGG-3' AS. Primers used for footprinting from -2750 bp to -777 bp are shown in table 1.

### Electrophoretic mobility shift assay (EMSA)

Oligonucleotides (both strands) corresponding to identified DNase I protected sites (table 2, 3, 5 and 6) were synthesized. For each site, one strand was end-labeled with [γ-^32^P] ATP using T_4 _polynucleotide kinase and annealed to its complementary unlabeled strand. Nuclear extracts (4–6 μg) were incubated with 20 fmoles of radiolabeled oligonucleotide duplex in 30 μl reaction containing 10 mM Tris-HCl (pH 7.5), 50 mM NaCl, 1 mM EDTA, 1 mM DTT, 5 % glycerol and 1.0 μg poly (dI-dC) for 20 minutes at room temperature. In competition experiments, 100-fold molar excess of unlabeled oligonucleotide duplexes were added during preincubation period. For antibody shift assay, C/EBPβ antibody (Santacruz, USA) was added after addition of nuclear extract and incubated at 4°C for 10 min. Free DNA and protein bound DNA was separated on 5% non-denaturing polyacrylamide gel in 0.5 X Tris-boric acid-EDTA (TBE). After electrophoresis, gels were blotted onto filter paper, dried and autoradiographed.

**Table 5:** Primers and oligonucleotide used for site- directed mutagenesis.

Mut Luc-3-1	GCTGgAGGCcTAGCTCTGTAGCAGAgTACAccCAAG
Mut Luc-3-2	CAgGGTCCTcGTTCcATtCCaG
Mut Luc-3-3	CCAGTgCAgACgAGCAAGCggCTGTATATgC
SP1-SMP	GCTCCCCCCCCCCGCCCCCCCCCAGGG
Mut-SP1	GCTCCtCCtCCtCGtCtCCCtCCAG
C/EBP-SMP	ACTGATGTACACATTCCTAAAACTGGC
Mut-C/EBP	ACTGgTGgACACAggCCTAggACTGGC

**Table 6:** Oligonucleotide of the three footprints between -513 to -352 bp used for EMSA.

**Footprint**	**DNase 1 protected region**	**Transcription factor**
Luc3-1	GGAGCTGGAGGCATAGCTCTGTAGCAGAATACATTCAAGGT	CdxA
Luc3-2	TTCAAGGTCCTAGTTCTATCCCAG	GATA-2
Luc3-3	AACTACCAGTACAAACAAGCAAGCAACTGTATACAT	SRY

### Construction of 5' and 3' -serially deleted SMP30 fragments and it's cloning into pGL3-Basic vector

To construct 5' -serially deleted SMP30 fragments Luc-SMP-XhoI reverse primer and the forward primers as mentioned in table 4, containing Kpn1 sites were used. The PCR amplification was carried out using step cycles (94°C for 30 s, 62°C for 30 s, 72°C for 30 s) for 35 cycles with a final extension at 72°C for 10 minutes. Then the PCR products were purified using QIAquick Gel Extraction Kit (Qiagen, USA). The serially deleted fragments and pGL3-Basic vector were digested with KpnI and XhoI enzyme (MBI Fermentas). The digested 5' -serially deleted fragments were then ligated into restriction enzyme digested pGL3-Basic vector using DNA ligase (USB, USA). The cloned fragments were then confirmed by vector specific PCR using RV and GL2 primer, and also by sequencing.

### Site-directed mutagenesis

Five to six bases of the transcription factor core binding site were mutated as shown in the table 5 (bases in small letter represents mutated base). For mutagenesis of transcription factors two sets of PCR were carried out using the following combination of primers: For Sp1: MutSp1 sense/Luc-SMP-XhoI antisense and Luc 5 sense/Mut Sp1 antisense; for C/EBP: Mut C/EBP sense/Luc-SMP-XhoI antisense and Luc 5 sense/Mut C/EBP antisense; for Mut Luc 3–1: Mut Luc3-1 sense/Luc-SMP-XhoI antisense and Luc-SMP-3 sense/Mut Luc 3–1 antisense; for Mut Luc 3–2: Mut Luc 3–2 sense/Luc-SMP-XhoI antisense and Luc-3 sense/Mut Luc 3–2 antisense; for Mut Luc 3–3: Mut Luc 3–3 sense/Luc-SMP-XhoI antisense and Luc-SMP-3 sense/Mut Luc 3–3 antisense. The PCR amplification was performed using step cycles (94°C for 1 min, 62°C for 30 s, 72°C 30 s) for 35 cycles with a final extension at 72°C for 10 minutes. Both the PCR products were purified using QIAquick Gel Extraction Kit. DNA was eluted using 30 μl of autoclaved deionised water. 5 μl of each PCR product was used as a template for the second round of PCR. For example: for construction of mutant Sp1 site: 5μl each of the PCR product Mut Sp1 sense/Luc-SMP-XhoI antisense and Luc-5 sense/Mut Sp1 antisense was used as template. For construction of mutant Sp1 and mutant C/EBP, Luc-5 and Luc-SMP-XhoI was used as forward and reverse primers. For construction of mutant Luc 3–1, Luc 3–2 and Luc 3–3, Luc-3 and Luc-SMP-XhoI was used as forward and reverse primers. PCR amplification was carried out using the same parameters as mentioned above. Then the PCR products were purified using QIAquick Gel Extraction Kit. The fragments with mutated transcription factor binding sites, having KpnI and XhoI restriction sites and pGL3-Basic vector were digested with KpnI and XhoI enzyme. The fragments were then ligated into restriction enzyme digested pGL3-Basic vector using DNA ligase. The cloned fragments were then confirmed by vector specific PCR using RV and GL2 primer, and the mutation was confirmed by sequencing.

### Transient transfection and luciferase assay

Transient transfections were carried out using RAG cells (mouse renal adenocarcinoma cell line) as SMP30 is also expressed in kidney. The cells were plated at a density of 2 × 10^5 ^cells per well in six well plates, 18 h before transfection. For transient transfection 2 μg of respective reporter plasmid DNA and 0.5 μg of pSV-β-gal control vector (Promega, USA) or 100 ng of pRL-TK control vector were cotransfected into cells using FuGENE reagent (Roche, USA). After 24 h post transfection, the cells were harvested, lysed, centrifuged and the lysate was used for luciferase assay using the luciferase assay system (Promega, USA). The colorimetric β-galactosidase assay was performed using β-Gal assay kit (Invitrogen, USA) and luciferase activity was divided by the β-galactosidase activity to normalize for transfection efficiency. For transfection of mutated constructs Renella was used as an internal control and dual luciferase assay was preformed to measure the luciferase reading as per manufacturer's instruction (Promega, USA). All the transfections were repeated in duplicates in three to five independent experiments. The number of independent experiments is being mentioned in respective figure legends.

## Abbreviations

SMP30: Senescence Marker Protein 30; EMSA: Electrophoretic Mobility Shift Assay; RLNE: Rat Liver Nuclear Extract.

## Authors' contributions

PCS and NM conceived the idea, designed and planned the experiments. BR and PCS wrote the manuscript. BR and RSP were involved in all experimentations. PRD was involved in designing and preparation of promoter-reporter constructs. All authors have analyzed the data and agreed with the final version of the manuscript.

## References

[B1] Fujita T (1999). Senescence marker protein-30 (SMP30): Structure and biological function. Biochem Biophys Res Commun.

[B2] Fujita T, Shirasawa T, Maruyama N (1996). Isolation and characterization of genomic and cDNA clones encoding mouse senescence marker protein-30 (SMP30). Biochim Biophys Acta.

[B3] Ishigami A, Kondo Y, Nanba R, Oshsawa T, Handa S, Kubo S, Akita M, Maruyama N (2004). SMP30 deficiency in mice causes an accumulation of neural lipids and phospholipids in the liver and shortens the life span. Biochem Biophys Res Commun.

[B4] Ishigami A, Fujita T, Handa S, Shirasawa T, Koseki H, Kitamura T, Enomoto N, Sato N, Shimosawa T, Maruyama N (2002). Senescence marker protein-30 knockout mouse liver is highly susceptible to tumor necrosis factor-alpha- and Fas-mediated apoptosis. Am J Pathol.

[B5] Kondo Y, Inai Y, Sato Y, Handa S, Kubo S, Shimokado K, Goto S, Nishikimi M, Maruyama N, Ishigami A (2006). Senescence marker protein 30 functions as gluconolactonase in L-ascorbic acid biosynthesis, and its knockout mice are prone to scurvy. Proc Natl Acad Sci USA.

[B6] Sar P, Rath B, Subudhi U, Chainy GB, Supakar PC (2007). Alterations in expression of senescence marker protein-30 gene by 3,3',5-triiodo-L: -thyronine (T(3)). Mol Cell Biochem.

[B7] Supakar PC, Fujita T, Maruyama N (2002). Identification of novel sequence-specific nuclear factors interacting with mouse senescence marker protein-30 gene promoter. Biochem Biophys Res Commun.

[B8] Liu Y, Michalopoulos GK, Zarnegar R (1994). Structural and functional characterization of the mouse hepatocyte growth factor gene promoter. J Biol Chem.

[B9] Mitchell PJ, Tjian R (1989). Transcriptional regulation in mammalian cells by sequence-specific DNA binding proteins. Science.

[B10] Yamada K, Tanaka T, Miyamoto K, Noguchi T (2000). Sp family members and nuclear factor-Y cooperatively stimulate transcription from the rat pyruvate kinase M gene distal promoter region via their direct interactions. J Biol Chem.

[B11] Lee YH, Williams SC, Baer M, Sterneck E, Gonzalez FJ, Johnson PF (1997). The ability of C/EBP beta but not C/EBP alpha to synergize with an Sp1 protein is specified by the leucine zipper and activation domain. Mol Cell Biol.

[B12] Turner ME, Martin C, Martins AS, Dunmire J, Farkas J, Ely DL, Milsted A (2007). Genomic and expression analysis of multiple Sry loci from a single Rattus norvegicus Y chromosome. BMC Genet.

[B13] Dewing P, Chiang CW, Sinchak K, Sim H, Fernagut PO, Kelly S, Chesselet MF, Micevych PE, Albrecht KH, Harley VR, Vilain E (2006). Direct regulation of adult brain function by the male-specific factor SRY. Curr Biol.

[B14] Dubin RA, Ostrer H (1996). Sry is a transcriptional activator. Mol Endocrinol.

[B15] Yuan X, Lu ML, Li T, Balk SP (2001). SRY interacts with and negatively regulates androgen receptor transcriptional activity. J Biol Chem.

[B16] Terzic N, Vujcic M, Ristic-Fira A, Krstic-Demonacos M, Milanovic D, Kanazir DT, Ruzdijic S (2003). Effects of age and dexamethasone treatment on glucocorticoid response element and activating protein-1 binding activity in rat brain. J Gerontol A Biol Sci Med Sci.

